# High resolution chromosome 3p, 8p, 9q and 22q allelotyping analysis in the pathogenesis of gallbladder carcinoma

**DOI:** 10.1038/sj.bjc.6600490

**Published:** 2002-08-12

**Authors:** I I Wistuba, A Maitra, R Carrasco, M Tang, P Troncoso, J D Minna, A F Gazdar

**Affiliations:** Department of Anatomic Pathology, Pontificia Universidad Catolica de Chile, Marcoleta 367, P.O. Box 114-D, Santiago, Chile; Department of Pathology, Johns Hopkins Medical Institutions, Baltimore, Maryland, MD 21209, USA; Hamon Center for Therapeutic Oncology Research, UT Southwestern Medical Center, Dallas, Texas, TX 75390, USA

**Keywords:** loss of heterozygosity, tumour suppressor gene, microsatellite marker, preneoplasia, microdissection

## Abstract

Our recent genome-wide allelotyping analysis of gallbladder carcinoma identified 3p, 8p, 9q and 22q as chromosomal regions with frequent loss of heterozygosity. The present study was undertaken to more precisely identify the presence and location of regions of frequent allele loss involving those chromosomes in gallbladder carcinoma. Microdissected tissue from 24 gallbladder carcinoma were analysed for PCR-based loss of heterozygosity using 81 microsatellite markers spanning chromosome 3p (*n*=26), 8p (*n*=14), 9q (*n*=29) and 22q (*n*=12) regions. We also studied the role of those allele losses in gallbladder carcinoma pathogenesis by examining 45 microdissected normal and dysplastic gallbladder epithelia accompanying gallbladder carcinoma, using 17 microsatellite markers. Overall frequencies of loss of heterozygosity at 3p (100%), 8p (100%), 9q (88%), and 22q (92%) sites were very high in gallbladder carcinoma, and we identified 13 distinct regions undergoing frequent loss of heterozygosity in tumours. Allele losses were frequently detected in normal and dysplastic gallbladder epithelia. There was a progressive increase of the overall loss of heterozygosity frequency with increasing severity of histopathological changes. Allele losses were not random and followed a sequence. This study refines several distinct chromosome 3p, 8p, 9q and 22q regions undergoing frequent allele loss in gallbladder carcinoma that will aid in the positional identification of tumour suppressor genes involved in gallbladder carcinoma pathogenesis.

*British Journal of Cancer* (2002) **87**, 432–440. doi:10.1038/sj.bjc.6600490 www.bjcancer.com

© 2002 Cancer Research UK

## 

Gallbladder carcinoma (GBC) is a relatively uncommon neoplasm which demonstrates considerable geographic and gender variation in incidence (Albores-Saavedra and Henson, 2000). For unknown reasons, it is one of the most frequent neoplasms in Chile, where it is the leading cause of cancer deaths in females (Lazcano-Ponce *et al*, 2001). It has been well established that invasive GBC is preceded by preneoplastic lesions, including dysplastic changes of the gallbladder epithelium (Albores-Saavedra and Henson, 2000). There is a very limited information about the molecular changes involved in the pathogenesis of GBC (Wistuba and Albores-Saavedra, 1999).

It is now well recognised that tumourigenesis is a multistep process resulting from the accumulation of sequential genetic alterations (Fearon and Vogelstein, 1990). In addition to oncogene activation, inactivation of tumour suppressor genes (TSGs) has been shown to play an important role in tumourigenesis (Fearon and Vogelstein, 1990). Allelic loss, manifested as loss of heterozygosity (LOH) at polymorphic loci, is recognised as a hallmark of tumour suppressor genes, whose other allele is inactivated by point mutations or by some other mechanism (Knudson, 1985). Thus, the finding of chromosomal regions with frequent incidence of LOH in a neoplasm suggests that those regions may harbour one or more TSGs.

Our recent genome-wide allelotyping analysis on GBC indicated that allelic losses at multiple sites of the genome are frequent in this neoplasm (Wistuba *et al*, 2001). We identified at least 21 chromosomal regions with frequent LOH in GBC, including 3p, 8p, 9q and 22q regions. Allelic losses at those chromosome sites have been implicated in the pathogenesis of several human neoplasms, suggesting that there probably are several different TSGs located in those chromosomal regions (Albrecht *et al*, 1994; Chaganti *et al*, 1995; Habuchi *et al*, 1995; El-Naggar *et al*, 1998; Miyakawa *et al*, 1998; Cheng *et al*, 1999; Wistuba *et al*, 1999a, 2000; Baffa *et al*, 2000; Choi *et al*, 2000; Lerman and Minna, 2000; Muscheck *et al*, 2000; Maitra *et al*, 2001).

The present study was undertaken to refine the regions of frequent allele loss involving chromosomes 3p, 8p, 9q and 22q in GBC identified with in our recent genome-wide allelotyping analysis (Wistuba *et al*, 2001). Using DNA extracted from archival paraffin-embedded tissue of GBC, and a panel of high-resolution polymorphic markers, we studied the shortest regions of overlapping LOH on chromosomes 3p, 8p, 9q and 22q. We also studied the role of those allele losses in GBC pathogenesis by examining LOH on those four chromosomal arms in gallbladder epithelia accompanying invasive GBC.

## MATERIALS AND METHODS

### Archival tumour specimens

Formalin-fixed paraffin-embedded material from 24 surgically resected primary invasive gallbladder carcinomas was obtained from cholecystectomy specimens resected between 1990 and 1998 at the Catholic University Medical School Hospital, Santiago, Chile, as part of an Institutional Review Board approved study. The patients consisted of 19 women and five men ranging in age from 51–85 years (mean age, 68 years). Six (25%) were well differentiated, eleven (46%) were moderately differentiated, and seven (29%) were poorly differentiated tubulo-papillary adenocarcinomas. The majority of the tumours were advanced GBCs (20 of 24 cases; 84%) with invasion of the gallbladder serosa; the remaining (four of 24 cases; 16%) were early GBCs, with invasion of the submucosa (one case; 4%) or muscularis propia (three cases; 12%) of the gallbladder.

### Normal epithelium and preneoplastic lesions accompanying gallbladder cancer

**Figure 1:**
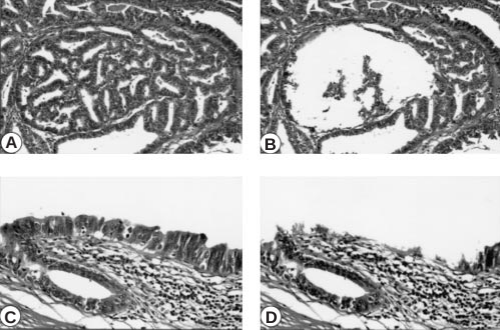
Representative example of the precise microdissection technique of invasive GBC (**A** and **B**) and high-grade dysplastic lesion (**C** and **D**) used in this study. Note that only tumour and epithelial cells were microdissected (**A**, **C**, before; **B**, **D**, after) while stromal tissue is intact.

Forty-five histologically discrete foci of non-invasive gallbladder epithelia were identified adjacent to 20 GBCs, each consisting of at least 1000 cells. These included 17 histologically normal epithelia and 28 high-grade dysplasias (Figure 1

). The dysplastic lesions were scored using published criteria for their histopathological identification in the gallbladder epithelium (Albores-Saavedra and Henson, 2000).

### Microdissection and DNA extraction

Serial 5 μm sections were cut from archival, formalin-fixed, paraffin-embedded tissues. Precise microdissection from archival paraffin-embedded tissues was performed under microscopic visualization using a micromanipulator, as described previously (Figure 1). From multiple serial sections of each case 4000 to 5000 sectioned tumour cells and 1000 gallbladder epithelial cells were microdissected, and DNA extracted as described. Dissected lymphocytes or normal stromal cells from the same slide were used as a source of constitutional DNA. DNA from at least 200 cells were used for each multiplex PCR reaction, as previously described (Wistuba *et al*, 1998). In order to circumvent the possibility of artifactual LOH occurring in DNA extracted from the relatively small non-tumour foci, fewer microsatellite markers were analysed to ensure comparable numbers of cells being used for the initial amplification reaction in all samples.

### Microsatellite DNA markers and PCR- LOH analysis

**Figure 2:**
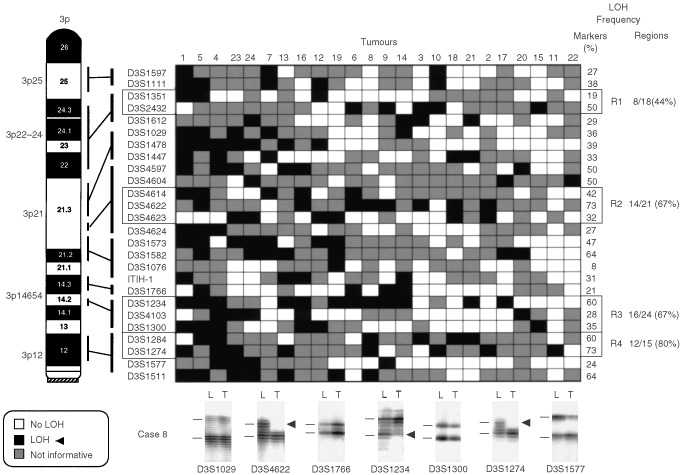
Patterns of chromosome 3p allele losses in gallbladder carcinoma. The cases have been arranged from left to right in decreasing order of chromosome 3p allele losses. Markers are placed in the predicted order from 3pter-cen. Four regions (*R1*–*R4*) having frequent allelic losses are shown. Lower panel: seven autoradiographs showing discrete allele loss at chromosome 3p in a microdissected GBC case (case 8) demonstrating loss and retention of neighbouring alleles. *L*, lymphocytes or normal stromal cells; *T*, microdissected invasive GBC. *Horizontal bars* on the left of the autoradiographs indicate the main allelic bands.

To evaluate LOH on GBC, we used primers flanking 81 microsatellite repeat polymorphisms spanning several 3p (*n*=26), 8p (*n*=14), 9q (*n*=29) and 22q (*n*=12) regions showing high frequencies of LOH in the genome-wide allelotyping analysis on GBC (Wistuba *et al*, 2001). The microsatellite markers tested are shown in Figures 2

,3

**Figure 3:**
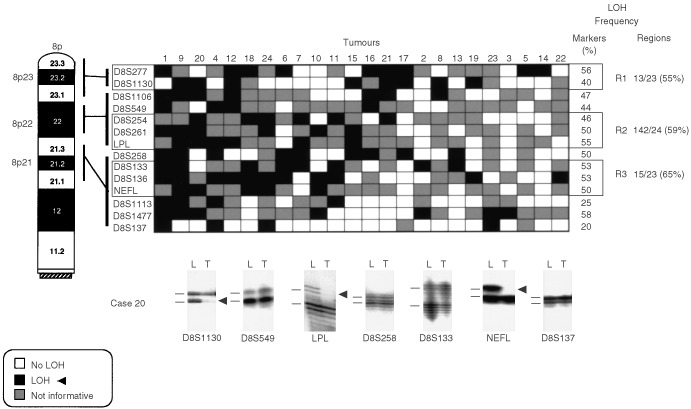
Patterns of chromosome 8p allele losses in gallbladder carcinoma. The cases have been arranged from left to right in decreasing order of chromosome 8p allelic losses. Markers are placed in the predicted order from 8pter-cen. Three regions (*R1*–*R3*) having frequent allelic losses are shown. Lower panel: seven autoradiographs showing discrete allele loss at chromosome 8p in a microdissected GBC case (case 20) demonstrating loss and retention of neighbouring alleles. *L*, lymphocytes or normal stromal cells; *T*, microdissected invasive GBC. *Horizontal bars* on the left of the autoradiographs indicate the main allelic bands.

,4

**Figure 4:**
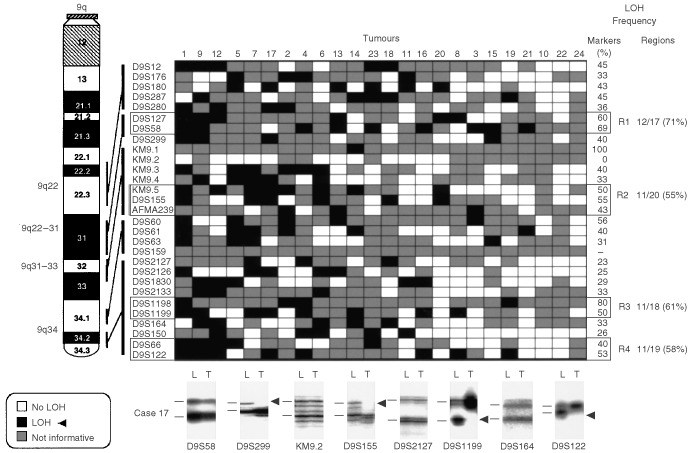
Patterns of chromosome 9q allele losses in gallbladder carcinoma. The cases have been arranged from left to right in decreasing order of chromosome 9q allele losses. Markers are placed in the predicted order from 9qcen-ter. Four regions (*R1*–*R4*) having frequent allelic losses are shown. Lower panel: eight autoradiographs showing discrete allele loss at chromosome 9q in a microdissected GBC case (case 17) demonstrating loss and retention of neighbouring alleles. *L*, lymphocytes or normal stromal cells; *T*, microdissected invasive GBC. *Horizontal bars* on the left of the autoradiographs indicate the main allelic bands.

,5

**Figure 5:**
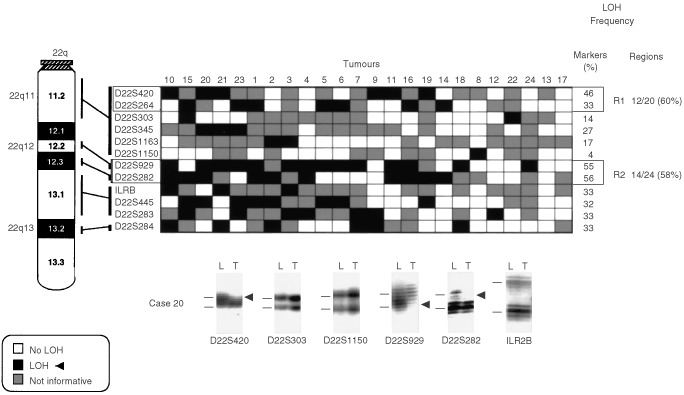
Patterns of chromosome 22q allele losses in gallbladder carcinoma. The cases have been arranged from left to right in decreasing order of chromosome 22q allele losses. Markers are placed in the predicted order from 22qcen-ter. Two regions (*R1*–*R2*) having frequent allelic losses are shown. Lower panel: six autoradiographs showing discrete allele loss at chromosome 22q in a microdissected GBC case (case 20) demonstrating loss and retention of neighbouring alleles. *L*, lymphocytes or normal stromal cells; *T*, microdissected invasive GBC. *Horizontal bars* on the left of the autoradiographs indicate the main allelic bands.

. The markers were selected from the Genome Database (http://www.gdb.org/). Subsets of markers (*n*=17) spanning a total of 12 chromosomal regions frequently deleted in invasive GBC (Table 1

**Table 1:**
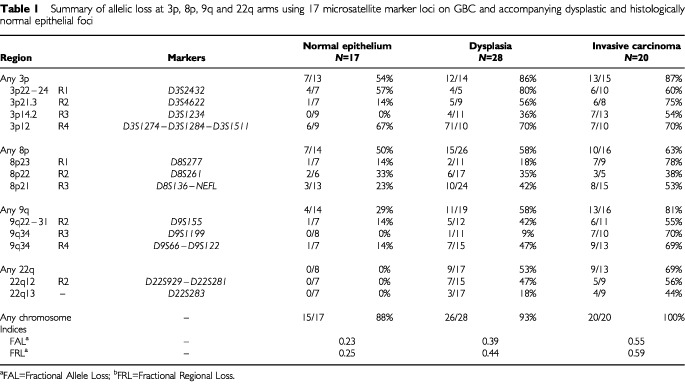
Summary of allelic loss at 3p, 8p, 9q and 22q arms using 17 microsatellite marker loci on GBC and accompanying dysplastic and histologically normal epithelial foci

) were used for the analysis of gallbladder epithelium (3p=6; 8p=4; 9q=4; and 22q=3). Because we used DNA extracted from archival paraffin-embedded tissues, the amplicon size was restricted to less than 250 bp. A two-round PCR strategy (multiplex PCR followed by uniplex PCR) was utilised to amplify each marker, as described previously (Wistuba *et al*, 1998). We optimised the PCR conditions to perform 11 multiplex PCRs containing all 81 markers utilised in this study. Each multiplex PCR contained six to eight microsatellite markers. The list of multiplex sets and specific optimization conditions we used are available upon request. A 10°C ‘touch-down’ PCR strategy was used spanning the primers annealing temperature followed by 25 cycles at the optimal annealing temperature. The final product was separated on a 6% denaturing polyacrylamide gel and subjected to autoradiography. LOH was scored by visual detection of complete absence of one allele of informative cases (Figures 2,3,4,5).

### Data analysis

The data were analysed using a series of *Microsoft Visual Basic* programmes specifically written for various computations or repetitive tasks in *Microsoft Excel*, including LOH frequencies, breakpoint frequencies, colour-coded formatting of LOH patterns, and clustering analysis, as described previously (Girard *et al*, 2000). In order to determine the sequence of genetic abnormalities in the multistep progression of GBC we utilised multiple microsatellite markers (*n*=17) to examine LOH at 12 chromosomal regions located on the four chromosomal arms examined. To determine whether the losses were progressive in the sequential development of GBC, we determined the frequency of loss of individual microsatellite markers (*n*=17) and chromosomal regions (*n*=12) using the Fractional Allele Loss (FAL) index and the Fractional Regional Loss (FRL) index, respectively. The FAL index is defined as the total number of microsatellite markers with LOH in an epithelial sample divided by the total informative markers in the corresponding normal DNA. The FRL index is defined as the total number of chromosomal regions with LOH divided by the total number of informative regions in the corresponding normal DNA.

Statistical analysis was performed using the nonparametric Wilcoxon and Fisher Exact tests. The cumulative binomial test was used to examine the likelihood that the occurrence of a particular event (loss of the same allele in the invasive carcinoma and an associated epithelial sample) occurs at a particular probability when observed in repeated trials. When the results are compared with a chance occurrence or nonoccurrence, the particular probability of comparison is 0.5. Probability values of *P*<0.05 were regarded as statistically significant.

## RESULTS

### Definition of regions of loss in GBC

Overall frequencies of allelic loss at any 3p (24 out of 24 cases, 100%), 8p (24 out of 24 cases, 100%), 9q (21 out of 24 cases, 88%), and 22q (22 out of 24 cases, 92%) sites were very high in GBCs. Most of the sites of allelic loss at all chromosomal arms examined were localised, and the extent of the partial allele losses was used to identify 13 discrete minimal regions of non-overlapping allele losses (four for 3p; three for 8p; four for 9q; and two for 22q). The patterns of LOH and frequencies of allelic loss at different critical regions identified are shown in Figures 2,3,4,5 and summarised in Table 2

**Table 2:**
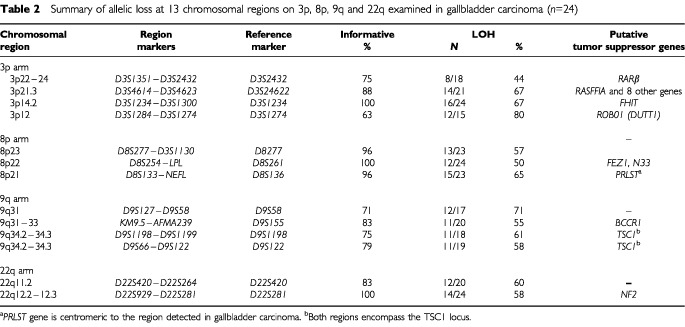
Summary of allelic loss at 13 chromosomal regions on 3p, 8p, 9q and 22q examined in gallbladder carcinoma (*n*=24)

. Representative autoradiographs for cases with partial loss of all four chromosomal arms demonstrating loss and retention of neighbouring alleles in GBCs are illustrated in Figures 2–5. Because artifacts resulting from PCR amplification may be mistaken for LOH, especially when minute amounts of input DNA are utilised, approximately 30% of examples of LOH, including many foci having distinct losses, were repeated for confirmation from newly microdissected material and identical LOH pattern were detected.

### Patterns of loss in GBC

Although most of the losses at all chromosomal arms examined were localised, the data demonstrate that in chromosomes 3p, 8p and 9q the most frequently observed pattern was loss of two or more regions. Thus, allelic loss of a single region in each of those chromosomes by itself was a relatively infrequent event. Two or more regions were lost in 84% at 3p, 73% at 8p, 63% at 9q, and 52% at 22q. Cluster analysis to examine if allelic loss at one chromosomal region was linked to changes at another region (Girard *et al*, 2000) did not reveal any concordance between markers from the same or different chromosomes.

### Allelic loss in normal and dysplastic gallbladder epithelia accompanying tumours

Allelic loss at one or more 3p, 8p, 9q and 22q regions was detected in the majority of the histologically normal (15 out of 17, 88%) and dysplastic (26 out of 28, 93%) foci examined (Table 1). The FAL (Fractional Allelic Loss) and FRL (Fractional Regional Loss) indices were calculated as an expression of the amount of allele loss, and an increasing severity of histological changes was characterised by a significant rise of both index means (*P*<0.001; Table 1).

**Figure 6:**
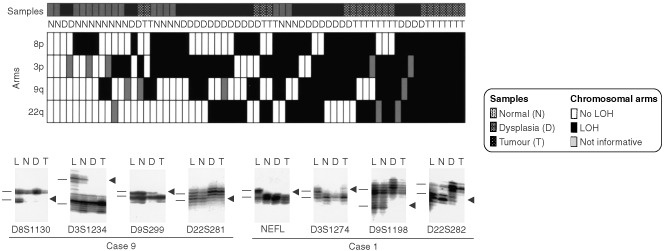
Summary of all allelotyping results by chromosome arms in the pathogenesis of GBC. A total of 69 specimens are shown, including all 24 invasive carcinomas and all 45 non-malignant epithelium (17 histologically normal appearing and 28 dysplastic epithelia) accompanying GBCs. The specimens have been sorted from left to right in ascending number chromosome arm allele losses. The sources of the various specimens are coded above the boxes and include normal epithelium (*N*), dysplasia (*D*) and tumour (*T*). Lower panel: eight autoradiographs showing discrete allele loss at chromosomes 3p, 8p, 9q y 22q in two GBCs and their accompanying non-malignant epithelium. *L*, lymphocytes or normal stromal cells; *N*, normal epithelium; *D*, dysplasia; *T*, microdissected invasive GBC. *Horizontal bars* on the left of the autoradiographs indicate the main allelic bands. Data analysis shows that allelic losses present in normal and dysplastic epithelia were not random. Analysis of informative samples for all four chromosome arms shows that most normal epithelia demonstrate 8p or 8p+3p losses and the majority of dysplasias have losses on 8p and/or 3p with 9q and/or 22q, sugesting a sequential model of genetic abnormalities that begins with 8p LOH and progresses through 3p, 9q and 22q losses.

The pattern of allelic loss was not random, and losses at one or more 3p (54%) and 8p (50%) regions were the most frequently detected abnormalities in histologically normal epithelium (Table 1). While 9q allelic loss was relatively frequent (29%) in normal epithelium, losses at 22q commenced at the dysplasia stage. For most of the chromosomes examined (3p, 8p and 22q), the differences between dysplasia and invasive carcinoma were modest and not significant. Data on individual epithelial and tumour samples are pooled in Figure 6

. As minute amounts of input DNA were utilised, all examples of LOH from the histologically normal epithelia and nearly half from the dysplasia were repeated for confirmation.

### Patterns of allelic loss in the pathogenesis of GBC

To determine the sequential molecular changes involved in the development of GBC, we analysed the pattern of allele losses detected in the GBC tumours and their accompanying normal and dysplastic epithelia. We considered only 14 tumours and 30 accompanying normal (*n*=11) and dysplastic (*n*=19) epithelial specimens that were informative for at least one marker in each chromosomal arm examined. From these specimens only a single dysplastic focus showed no LOH at any chromosomal arm and was excluded from this analysis. Three patterns of allelic loss were discerned in the 30 histologically normal, dysplastic and neoplastic foci: (a) Early pattern, with only 8p loss or 8p accompanied with 3p. (b) Intermediate pattern, with only 9q loss or 9q accompanied with 3p and/or 8p. (c) Advanced pattern, with 22q loss accompanied with 8p+3p or 8p+9q or 8p+3p+9q. Of interest, normal gallbladder epithelium had only early (55%) and intermediate (45%) patterns, while tumours had advanced (71%) and intermediate (29%) patterns. Dysplastic gallbladder epithelia demonstrated the entire spectrum of patterns, including the advanced (56%), intermediate (30%) and early (14%).

### Allele specific mutations* vs* potential clonal relationship of epithelial foci

Previous studies in gallbladder carcinoma and other neoplasms demonstrated that at any one locus, loss of parental alleles was not random, and that there was a strong tendency for the identical allele to be lost in all non-neoplastic and neoplastic foci examined (Wistuba *et al*, 1995, 1999b). We refer to this phenomenon as allele specific mutation (ASM) (Wistuba *et al*, 1995, 1999b). We determined the frequencies of ASM in the 61 epithelial and tumoural foci demonstrating one or more sites of allelic loss in 20 GBC cases. For all 146 comparisons involving 17 microsatellite markers, the same parental allele was lost in 128 (88%). The possibility that this occurred by chance alone is extremely remote as tested by the cumulative binomial test (*P*=5.3×10^−22^).

Because in the GBC there is a close morphological relationship between invasive carcinoma and its dysplastic lesions (Albores-Saavedra and Henson, 2000) we then examined the possibility that cancer and non-malignant gallbladder epithelia were clonally related, using the specific alleles lost to mark the different foci in individual cases. Possible evidence of a clonal relationship (through sequential changes) were present only in 10 (16%) out of 61 foci examined. In most (84%) of the normal and dysplastic epithelia the patterns of allele loss suggested that they arose as independent clones. However, we cannot exclude the possibility that the lesions represent subclones from a precursor clone having some initial molecular change, which we did not examine. Despite this lack of clonal relationship ASM was still significantly detected even in the analysis of clonally unrelated foci (104 of 123 comparisons, 85%; *P*=9.1×10^−26^).

### Microsatellite instability (MSI) in GBC pathogenesis

We detected a relatively high frequency of MSI at one or more 3p, 8p, 9q and 22q chromosomal loci in one of 13 (8%) of histologically normal foci, in four of 54 (7%) dysplastic foci and in six of 12 (50%) invasive carcinomas examined (data not shown).

## DISCUSSION

Our recent genome-wide allelotyping analysis indicated that allelic losses at multiple sites of the genome are frequent in GBC, and indicated for the first time that in addition to 3p and 8p, LOH on 9q and 22q may also play a role in the pathogenesis of this neoplasm (Wistuba *et al*, 2001). We identified at least 21 chromosomal regions with frequent LOH in GBC, including sites on 3p, 8p, 9q and 22q, suggesting that those chromosome regions may harbour putative TSGs that are inactivated in the development of this tumour. This previous study was a low-density screen in order to elucidate chromosomal hot spots for subsequent detailed analysis.

The present study with high-density allelotyping have confirmed and extended the previous findings. The overall frequencies of allelic loss at any 3p (100%), 8p (100%), 9q (88%), and 22q (92%) sites were very high in our tumours. Overall, thirteen distinct sites of frequent allele loss in GBC at chromosome 3p, 8p, 9q and 22q (summarised in Table 2) were detected in the present study, and the majority of these have not previously been described in this neoplasm. The location of these sites was based on the determination of the minimal region of loss that is defined by the occurrence of ‘breakpoints’ surrounding regions of frequent LOH. These sites are likely to represent TSG regions that are lost in GBC and warrant further investigation.

As a result of our detailed allelotyping 3p analysis on GBC, we were able to identify multiple areas of discontinuous LOH and four distinct 3p regions (3p22-24; 3p21.3; 3p14.2; and 3p12) with frequent allelic loss in this tumour. Several candidate TSGs have been detected in those 3p regions with frequent allele losses in GBC. One candidate in the 3p22-24 region is the retinoic acid receptor-beta (*RAR*β) gene (Virmani *et al*, 2000). Another candidate gene, the fragile histidine triad (*FHIT*) gene, spans the *FRA3B* fragile site at 3p14.2 (Huebner *et al*, 1998). A new candidate TSG, *ROB01* (*DUTT1*), has been cloned residing in the U2020 3p12 deletion region at marker *D3S1274* (Sundaresan *et al*, 1998). Currently two distinct 3p21.3 regions are under study because of the existence of multiple homozygous allele loss in lung and breast cancer cell lines (Lerman and Minna, 2000). One of those was tested in this report by markers *D3S4614* and *D3S4622*, and represents a minimal region of frequent allele losses (67%) in our GBC cases. Recently, a new candidate TSG located in this region, the human *RAS* effector homologue (termed *RASSF1A*) gene, has been shown to have tumour suppressing function and undergoes epigenetic inactivation in several cancers (Dammann *et al*, 2000; Burbee *et al*, 2001; Dreijerink *et al*, 2001).

Our detailed allelotyping analysis of the 8p21-23 region demonstrated three distinct regions with frequent allelic loss on GBC, namely 8p23, 8p22, and 8p21. The 8p21 region, defined by the same markers tested in our GBCs, has been described with frequent allele loss in other tumour types (Trapman *et al*, 1994; Farrington *et al*, 1996; Baffa *et al*, 2000). The platelet-derived growth factor receptor-like gene *(PRLTS*) candidate TSG is centromeric to our 8p21 minimal region (Fujiwara *et al*, 1995). The 8p22 region identified in our GBCs spans the *FEZ1/LZTS1* gene, a candidate TSG, whose expression is altered in multiple human tumours (Ishii *et al*, 1999; Cabeza-Arvelaiz *et al*, 2001). The *N33* gene at 8p22 was found to be silenced in several cancer cells, although no point mutations have been identified (Bookstein *et al*, 1997). Allele losses at the telomeric 8p23 region, shown to be frequent in our gallbladder tumours, has been previously described on head and neck, hepatocellular and prostate carcinomas (Perinchery *et al*, 1999; Pineau *et al*, 1999; Ishwad *et al*, 1999). However, no candidate genes have been reported on this region.

At 9q, four different regions of frequent allele losses in GBC were detected, namely 9q31, 9q31-33, and two sites at 9q34.2-34.3. While the 9q31 minimal region has not been previously described in other tumour types, allele losses at the 9q32-33 and 9q34 regions have been frequently reported in other neoplasms (Chaganti *et al*, 1995). The 9q32-33 minimal region has been intensively studied in transitional cell carcinoma of the urinary bladder (Habuchi *et al*, 1998, 2001), being designated as DBC1 locus (for deleted in bladder cancer gene 1). A novel candidate gene for this region has been identified and designated as *DBCCR1* (Habuchi *et al*, 1998, 2001). At least two regions of minimal allele losses involving the telomeric 9q34.2-34.3 region were detected in GBC. These regions encompass one of the loci identified for the tuberous sclerosis gene (TSC) (van Slegtenhorst *et al*, 1997).

We have detected two distinct sites of frequent allelic loss in GBC, 22q11.2 and 22qq12.2-q12.3 regions. Both regions have been described with frequent allele losses in other neoplasms. The centromeric 22q11.2 region has been previously reported in hepatocellular (Takahashi *et al*, 1993) and breast carcinomas (Allione *et al*, 1998); however, no candidate TSG has been reported at this site. The 22q12.2-q12.3 region encompasses the neurofibromatosis type 2 (*NF2*) gene, a candidate TSG at this region (Rouleau *et al*, 1993). Because *NF2* gene mutations are rarely seen in epithelial tumours with high frequency of allele losses at the *NF2* gene locus (Takahashi *et al*, 1993; Englefield *et al*, 1994; Allione *et al*, 1998; Miyakawa *et al*, 1998), an additional TSG involved in tumourigenesis could be located at this region.

In several neoplasms it has been established that multiple sequential genetic changes are associated with the development of invasive tumours. However, few investigators have focused in the genetic abnormalities involved in the development of gallbladder carcinoma. We and others (Wistuba *et al*, 1995; Chang *et al*, 1999) have reported a high incidence of allelic loss at several chromosomal regions (17p-*TP53*, 9p21-*p16/CDKN2*, and 5q22-*APC/MCC* region) occurring early during the sequential pathogenesis of GBC. The present findings of frequent chromosome 3p, 8p, 9q and 22q allele losses in non-malignant gallbladder epithelia confirm and greatly extend the findings that molecular changes commence early (in histologically normal epithelium) during the sequential pathogenesis of GBC. Our major findings regarding the molecular pathogenesis of GBC are: (1) molecular changes preceded the onset of histologically recognisable changes and 88% of the normal histologically normal foci have allele loss at one or more chromosomal regions examined. (2) There was a progressive increase of the overall LOH frequency expressed by the FAL and FRL indices, with increasing severity of histopathological changes. The development of epithelial cancers requires multiple mutations (Fisher, 1958), and the stepwise accumulation of these mutations may represent an inherent mutator phenotype (Loeb, 1991). Thus, it is likely that those preneoplastic lesions that have accumulated multiple mutations are also the ones at higher risk for progression to invasive cancer. (3) Allelic losses present in normal and dysplastic epithelia were not random. The most frequent regions of allelic loss at normal epithelium occurred at 3p and 8p. While 9q allelic losses were present mainly in dysplastic lesions, losses at 22q were only detected in advanced lesions (dysplasia and invasive carcinoma). By examining all our material for 3p, 8p, 9q and 22q allele loss, we propose a sequential model of genetic abnormalities that begins with 8p LOH and progresses through 3p, 9q and 22q. 4) The same parental allele was frequently lost (88% of comparisons) in non neoplastic lesions as in the corresponding invasive carcinomas. We refer to this phenomenon as allele specific mutation (ASM). We have documented this phenomenon in several neoplasms (Wistuba *et al*, 2000; Maitra *et al*, 2001), including GBC (Wistuba *et al*, 1995). The mechanism underlying ASM remains unknown. Possibilities include the following: (a) seeding of multiples sites in the gallbladder epithelium with a common progenitor clone; (b) inhereted differences (e.g., polymorphism) in alleles that determine their susceptibility to loss; and, (c) potential epigenetic differences in alleles that arise during development (e.g., methylation) that might predispose one to preferential loss. Since there is a close morphological relationship between invasive carcinoma and dysplastic lesions (Albores-Saavedra and Henson, 2000), we examined the possibility that the ASM phenomenon represented clonal relationship due to a seeding or spread in the gallbladder epithelium from a common progenitor clone. However, our findings that most (84%) of the normal and dysplastic gallbladder epithelia accompanying invasive tumours arose as independent clones suggest that ASM has a different basis than clonality in GBC, such as inhereted susceptibility to loss or potential epigenetic differences that might predispose to preferential allele loss.

Alterations in microsatellite size, microsatellite instability (MSI), are present in many cancers, including GBC (Wistuba *et al*, 1995), and reflect a form of genetic instability. We found MSI in 50% of the invasive GBC tumours and in lesser percentages of histologically normal and dysplastic foci. The mechanism involving MSI in GBC need to be further studied.

In summary, allele losses of chromosome 3p, 8p, 9q and 22q regions are frequent in GBC. Our data identifies 13 distinct regions of loss on those chromosomal arms, many of which harbour one or more candidate TSGs, which may play a role in GBC pathogenesis. These regions have previously been reported to be frequently lost in several human cancers, suggesting that may harbour TSGs whose inactivation may be critical to the process of tumourigenesis. In addition, our findings in the non-malignant gallbladder epithelium indicate that multiple, non-random and sequential allele specific abnormalities commence early in the multistage pathogenesis of GBC. These findings should be useful for the identification of the TSGs involved in the pathogenesis of GBC, with the potential for defining molecular markers for early detection as well as for the development of gene therapy strategies of this highly malignant neoplasm.
